# The relationship between the dislocations and microstructure in In_0.82_Ga_0.18_As/InP heterostructures

**DOI:** 10.1038/srep35139

**Published:** 2016-10-11

**Authors:** Liang Zhao, Zuoxing Guo, Qiulin Wei, Guoqing Miao, Lei Zhao

**Affiliations:** 1 Key Lab of Automobile Materials Ministry of Education, College of Materials Science and Engineering, Jilin University, Changchun, 130025, P.R. China; 2 State Key Laboratory of Luminescence and Applications, Changchun Institute of Optics, Fine Mechanics and Physics, Chinese Academy of Sciences, Changchun, 130033, P.R. China

## Abstract

In this work, we propose a formation mechanism to explain the relationship between the surface morphology (and microstructure) and dislocations in the In_0.82_Ga_0.18_As/InP heterostructure. The In_0.82_Ga_0.18_As epitaxial layers were grown on the InP (100) substrate at various temperatures (430 °C, 410 °C and 390 °C) using low pressure metalorganic chemical vapor deposition (LP-MOCVD). Obvious protrusions and depressions were obseved on the surface of the In_0.82_Ga_0.18_As/InP heterostructure because of the movement of dislocations from the core to the surface. The surface morphologies of the In_0.82_Ga_0.18_As/InP (100) system became uneven with increasing temperature, which was associated with the formation of dislocations. Such research investigating the dislocation of large lattice mismatch heterostructures may play an important role in the future-design of semiconductor films.

As a typical III-V compound, In_x_Ga_1−x_As is one of the most important semiconductor materials^1^,^2^. Because of their excellent photoelectric properties, III-V compound films have been widely used in t infrared detectors^3^,^4^, solar cells^5^,^6^, transistors^2^,^7^, optical switches^8^ and optical fibre communications devices^9^. Compared to other In_x_Ga_1−x_As films, films of high-In-content semiconductor, such as In_0.82_Ga_0.18_As, which has a long cut-off wavelength (more than 2 μm) in spectroscopic applications, have attracted more attention^10^,^11^.

The methods used to prepare semiconductor films strongly influence their dislocation density and photoelectric properties, thus, various epitaxial growth technologies such as MBE^12^,^13^, SPE^14^, PVD^15^ have been explored t in recent years to obtain high performance semiconductor thin films. Among these main thin-film preparation method, metalorganic chemical vapor deposition (MOCVD) has been widely used in the preparation of In_x_Ga_1−x_As materials since 1968^16^,^17^,^18^. InP and GaAs are common substrates used in the fabrication of heterostructures. The lattice mismatch between the In_0.82_Ga_0.18_As epitaxial layer and substrate strongly affects the performance of the In_0.82_Ga_0.18_As films. The lattice mismatch in the In_0.82_Ga_0.18_As /InP heterostructure is 2%, whereas that in In_0.82_Ga_0.18_As /GaAs heterostructure system is greater than 5.6%^19^. Two-step growth^20^ or the insertion of step-graded buffer layers between the substrate and the epitaxial layer^21^ are common and critical approaches used to improve the quality of the epitaxial layers.

In previous studies, researchers invoked the Frank-van der Merwe, Stranski-Krastanov and Volmer-Weber growth-mode models (2D-to-3D growth-mode transition) to discuss the formation mechanism of films^22^,^23^,^24^,^25^. These models directly explain the growth process of thin films. However, the literature still contains little intuitionistic explanation about the relationship between the surface morphology and the dislocations. In our previous report, we only analysed the dislocation types (60° and 90° dislocations) at the interface in detail (the In_0.82_Ga_0.18_As films were prepared at 430 °C)^26^. As a consequence, the strain in the In_0.82_Ga_0.18_As/InP (100) interface was incompletely relaxed because of the formation and multiplication of misfit dislocations (MDs). Various defects including stacking faults as well as 60° and 90° threading dislocations were identified in the region near the interface, and the plastic relaxation of the strained heterostructures was obtained by the creation of MDs. The styles and the formation of the dislocations near the interface in the heterostructures have been analysed systematically in previous reports^27^,^28^,^29^,^30^.

In this work, we focused on the formation mechanism to explain the relationship between the surface morphology (and microstructure) and dislocations of In_0.82_Ga_0.18_As/InP heterostructure. We describe the relationship between the protrusions and depressions on the surface of the epitaxial layers. Furthermore, the movement of dislocations is further investigated. Additionally, we calculate the dislocation density in the epitaxial layers to explore the influence of the movement of dislocations on the surface morphology and the microstructure of epitaxial layers. In this paper, we emphasize our development of a formation mechanism to explain the relationship between the surface morphology (and microstructure) and the dislocations of In_0.82_Ga_0.18_As/InP heterostructure. To explain our model more clearly, we have included two additional experiments (at 390 °C and 410 °C) to provide better contrast with previous results.

## Results

**Figure 1:**
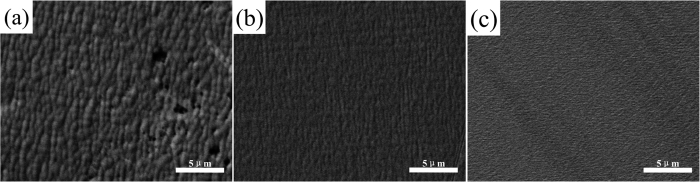
Surface morphology figures of the In_0.82_Ga_0.18_As layer obtained at different growth temperatures: (**a**) 430 °C; (**b**) 410 °C; (**c**) 390 °C.

The In_0.82_Ga_0.18_As/InP specimens were grown using the MOCVD (see Methods for details). The samples grown at different temperatures (430 °C, 410 °C and 390 °C) are labelled as sample A, sample B, and sample C, respectively. The surface morphologies of In_0.82_Ga_0.18_As layers obtained at different growth temperatures were examined by SEM; the results are shown in Fig. 1(a–c). Numerous small protrusions and depressions were observed on the surface of sample A, as shown in Fig. 1(a), and these protrusions were noticeably smaller on samples B and C, as shown in Fig. 1(b,c), respectively. Our observations demonstrate that the surface morphology became smooth as the preparation temperature was descread. To elucidate the formation of the protrusions and depressions more clearly, we characterized the three specimens by transmission electron microscopy (TEM).

**Figure 2:**
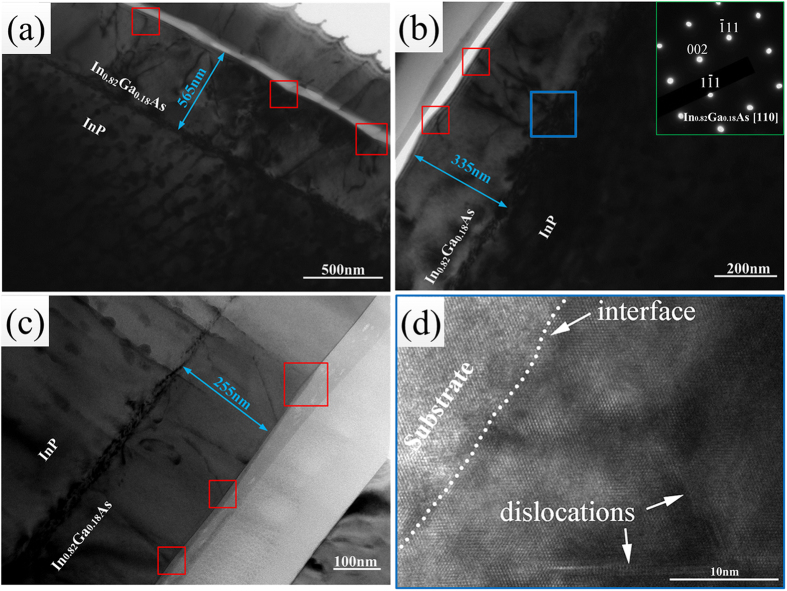
Cross-sectional views of the In_0.82_Ga_0.18_As/InP epitaxial layers for the [110] zone which were deposited simultaneously and at different preparation temperatures: (**a**) 430 °C; (**b**) 410 °C; (**c**) 390 °C.

Figure 2 shows the [110] cross-section fragments of the three samples characterized by TEM. For samples A, B and C, the growth rates of the In_0.82_Ga_0.18_As epitaxial layer were 300, 187 and 142 nm/h at the same growth time, respectively. For samples A, B and C, shown in Fig. 2(a–c), respectively, the width of the protrusions (depressions) were approximately 520, 200 and 110 nm, whereas the heights were 90, 55 and 10 nm, respectively. These results are consistent with the SEM images in Fig. 1(a–c), respectively. The cross-section of the protrusions and the depressions on the surface arranged according to a definite principle of: depression-protrusion-depression (or protrusion-depression-protrusion); the dislocations gathered in the protrusions (depressions) shown in the red squares on the surface. The numbers of dislocations at the surface especially in the protrusions (depressions) decreased with decreasing temperature. Fig. 2(d), with blue borders, is a high resolution image at the interface of sample B (the blue square in Fig. 2(b)). Moreover, numbers of dislocations were observes at the interface because of the lattice mismatch of 2% between the In_0.82_Ga_0.18_As epitaxial layer and the InP substrate of each sample, as shown in Fig. 2(d).

## Discussion and Conclusion

**Figure 3:**
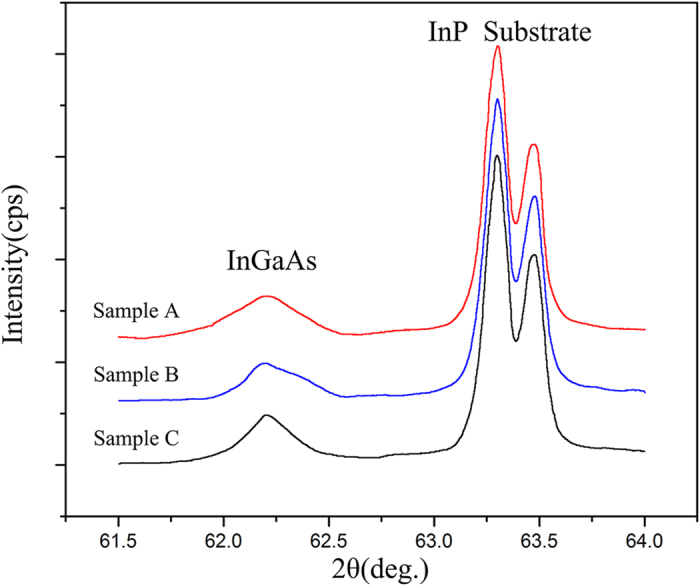
The XRD patterns of the InGaAs epitaxial layer of samples A–C.

**Figure 4:**
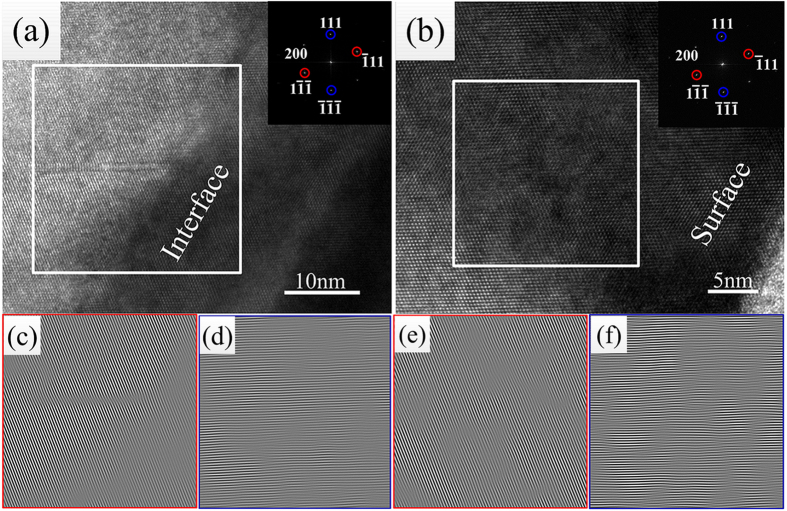
High-resolution electron microscope images and magnified inverse fast Fourier transform (IFFT) images of sample B from different regions: (**a**) high-resolution images at the interface; (**b**) high-resolution images at the surface; (**c**,**d**,**e**,f) IFFT images for plane groups.

On the basis of the aforementioned results, we propose that the surface structures are associated with the movement of the dislocations; this hypothesis can be strongly supported by calculation of the dislocation density. Usually, the full width at half maximum (FWHM) and the magnified inverse fast Fourier transform (IFFT) are used to calculate the dislocation density. The FWHM of the In_0.82_Ga_0.18_As epitaxial layer is an important parameter for crystalline structures examined using X-ray diffraction (XRD), according to the following formula:


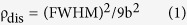


where b is a constant associated with the lattice parameter of In_0.82_Ga_0.18_As^31^, thus, as the value of the FWHM increases, the dislocation density increases. The values are summarized in Table. 1. The XRD patterns of the InGaAs epitaxial layer and InP substrates for samples A–C are shown in Fig. 3. However, with this method, the FWHM value only reflects the average dislocation density of the epitaxial layers; we therefore calculate the dislocation density of the surface and the interface using the magnified inverse fast Fourier transform (IFFT) technique. We define the dislocation density according to the following formula:


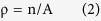


where n is the number of dislocations and A is the area. On the basis of this method, the dislocation density of the interface (or the surface) can be obtained. As shown in Fig. 4, we obtained two groups of (111) half-planes on the basis of IFFT, as shown in the magnified regions of the red and blue squares, we then used Equation (2) to determine the values of the dislocation density and average the values. Using a large number of high-resolution electron microscope images, we calculated the dislocation densities of the different regions; the results are collected in Table. 1. Furthermore, from Table. 1, the dislocation density at the interface was obviously larger than that in the epitaxial layer. As the growth temperature was lowered, the dislocation densities of both the interface and the surface decreased, consistent with the trend obtained from the Equation (1). As evident for sample A, the value of ρ_s_/ρ_i_ was 45.8–65.5%, whereas the values were 23.3–47.8% and 16.2–28.1% for sample B and sample C, respectively. These results indicate that at a higher temperature, more dislocations form and move to the surface. This observation is also consistent with the results shown in Fig. 2(a–c).

**Figure 5:**
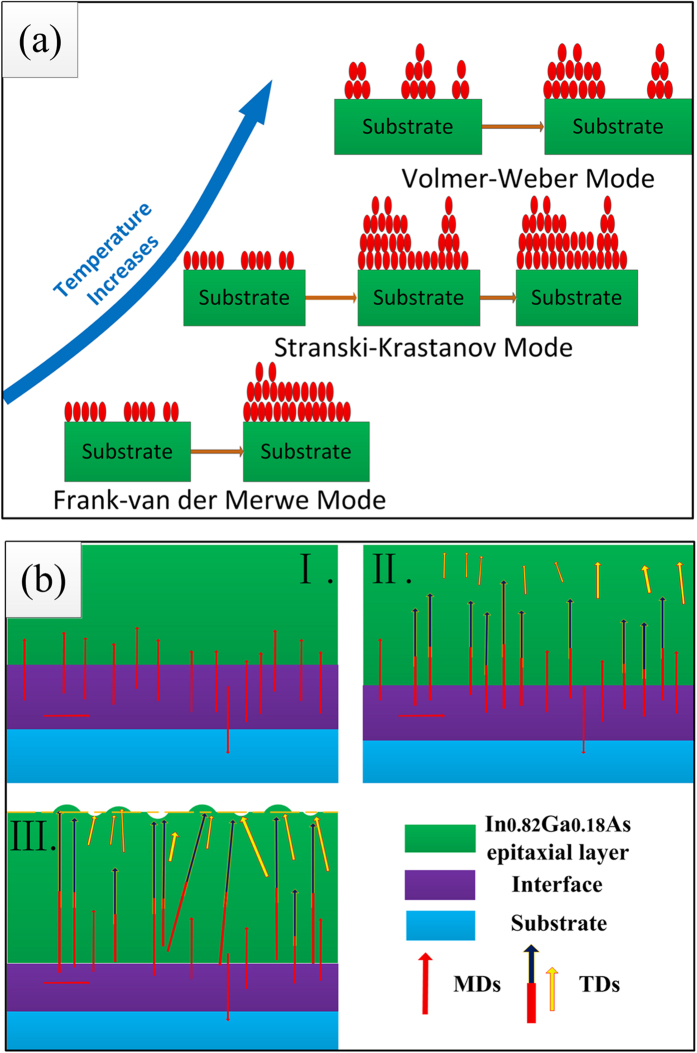
The two models of the different growth modes for the formation mechanism of films.

In previous reports, researchers have applied an acknowledged model in which the thin films grow via the Frank-van der Merwe, Stranski-Krastanov and Volmer-Weber growth mode (2D-to-3D growth-mode transition) to directly explain the formation mechanism of the films, as shown in Fig. 5(a). In this model, as the temperature increases, the diffusion of the atoms becomes much stronger and the strain increases; the growth of the films thus transitions from 2D growth into 3D growth to release the strain. On the basis of this model and the aforementioned analysis in this work, we here establish a simple model in which the dislocation motion in the films explains the formation mechanism of surface morphology, in cases not explained by the aforementioned model. As shown in Fig. 5(b), at the protrusions (or depressions) on the surface of heterostructure film, the dislocations lines generate together, leading to a poor surface. During the film formation process, misfit dislocations (MDs, red lines) are first formed because of the lattice mismatch in In_0.82_Ga_0.18_As/InP heterosystem. Second, as the film thickness increases, interaction dislocations incline towards the surface to form threading dislocations (TDs): some TDs form that depend on the dislocations’ glide plane as segments (the yellow lines) and some form from the Frank–Read source (black lines)^26^. These TDs then propagate to the surface and result in the formation of protrusions (or depressions). At a higher growth temperature, the atomic motion becomes much stronger, and more defects, especially dislocations, are formed. When the film growth rate becomes faster, a thicker film is obtained; in a thicker film, the strain increases, resulting in the generation of more dislocations on the surface. Finally, larger and more protrusions (depressions) are formed.

In summary, the quality of the surface morphology improves and the rough surface becomes smooth with decreasing temperature. We studied the relationship between the surface morphology and the microstructure of the epitaxial layer about the dislocation motion at different temperatures. We then devised a simple model to explain the formations of films and the protrusions (or depressions) on their surface. As the film growth progresses, interaction dislocations incline form threading dislocations (TDs): some TDs form that depend on thedislocations’ glide plane as segments and some form from the multiplication of the misfit dislocations (MDs). These TDs then generate on the surface and result in formation of the protrusions (or depressions). This model has the potential to be very influential in demonstrating how to use dislocations (or other defects) in the surface to improve the performance of films through surface treatments. Although the quality of our films (in terms of dislocation density) is not better than that of the films fabricated with a buffer layer, we are able to design and study how to use the dislocation of the surface to improve the surface morphology and properties, and we are able to take the epitaxial layers in this paper as the buffer layers, which represents an improvement upon the two-step growth method. Our research has the potential to play an important role in the design of semiconductor films (especially using the two-step growth method) and dislocation analysis of large lattice mismatch systems in the future.

## Methods

Sample preparation. The In_0.82_Ga_0.18_As epitaxial layers on InP(100) were grown by low-pressure MOVCD (AIXTRON 200/4) at three different temperatures (430 °C, 410 °C and 390 °C), because the pyrolysis temperature of trimethylgallium (TMGa) is higher than that of Trimethylindium (TMIn). At lower temperatures, controlling indium-gallium ratio at the same growth time (90 min) to synthesize the In_0.82_Ga_0.18_As is difficult. In particular, at 370 °C, the TMGa cannot be broken down^32^. TMGa, TMIn and 10% arsine (AsH_3_) in H_2_ were used as precursors. Palladium-diffused hydrogen was used as a carrier gas. The substrates on the graphite susceptor were heated under inductively coupled radio frequency power. The reactor pressure was maintained at 1 × 10^4^ Pa. The growth time was 90 min, and the growth rates of the In_0.82_Ga_0.18_As epitaxial layer were 300, 187 and 167 nm/h, respectively.

Characterization techniques. A high-resolution X-ray diffractometer (D8, Bruker) was used for the FWHM measurements to investigate the crystalline quality of the epitaxial layers. The surface morphology of the In_0.82_Ga_0.18_As/InP (100) system was detected by a scanning electron microscope (EVO-18, ZEISS). The samples for TEM observations were thinned manually and made electron-transparent by ion-milling using a Leica RES101 ion polishing system. A transmission electron microscope (JEM-2100F, JEOL) operated at 200KV was used for TEM observations; high resolution transmission electron microscopy (HRTEM) was used to observe [110] cross-section samples.

## Additional Information

**How to cite this article**: Zhao, L. *et al.* The relationship between the dislocations and microstructure in In_0.82_Ga_0.18_As/InP heterostructures. *Sci. Rep.* **6**, 35139; doi: 10.1038/srep35139 (2016).

## Figures and Tables

**Table 1:** Variation of temperature, dislocation density of the interface (ρ_i_), dislocation density of the surface (ρ_s_) and the value of ρ_s_/ρ_i_.

Sample	Temperature (°C)	FWHM (degree)	ρ_i_ (cm^−2^)	ρ_s_ (cm^−2^)	ρ_s_/ρ_i_
A	430	0.489	4.8 × 10^12^	2.5 × 10^12^	45.8–65.5%
B	410	0.343	3.7 × 10^12^	1.5 × 10^12^	23.3–47.8%
C	390	0.328	2.9 × 10^12^	0.7 × 10^12^	16.2–28.1%
